# Can We Set Aside Previous Experience in a Familiar Causal Scenario?

**DOI:** 10.3389/fpsyg.2020.578775

**Published:** 2020-11-30

**Authors:** Justine K. Greenaway, Evan J. Livesey

**Affiliations:** School of Psychology, The University of Sydney, Camperdown, NSW, Australia

**Keywords:** associative learning, causal learning, prediction error, heuristics, reasoning, attention

## Abstract

Causal and predictive learning research often employs intuitive and familiar hypothetical scenarios to facilitate learning novel relationships. The allergist task, in which participants are asked to diagnose the allergies of a fictitious patient, is one example of this. In such studies, it is common practice to ask participants to ignore their existing knowledge of the scenario and make judgments based only on the relationships presented within the experiment. Causal judgments appear to be sensitive to instructions that modify assumptions about the scenario. However, the extent to which prior knowledge continues to affect competition for associative learning, even after participants are instructed to disregard it, is unknown. To answer this, we created a cue competition design that capitalized on prevailing beliefs about the allergenic properties of various foods. High and low allergenic foods were paired with foods moderately associated with allergy to create two compounds; high + moderate and low + moderate. We expected high allergenic foods to produce greater competition for associative memory than low allergenic foods. High allergenic foods may affect learning either because they generate a strong memory of allergy or because they are more salient in the context of the task. We therefore also manipulated the consistency of the high allergenic cue-outcome relationship with prior beliefs about the nature of the allergies. A high allergenic food that is paired with an inconsistent allergenic outcome should generate more prediction error and thus more competition for learning, than one that is consistent with prior beliefs. Participants were instructed to either use or ignore their knowledge of food allergies to complete the task. We found that while participants were able to set aside their prior knowledge when making causal judgments about the foods in question, associative memory was weaker for the cues paired with highly allergenic foods than cues paired with low allergenic foods regardless of instructions. The consistency manipulation had little effect on this result, suggesting that the effects in associative memory are most likely driven by selective attention to highly allergenic cues. This has implications for theories of causal learning as well as the way causal learning tasks are designed.

## Introduction

Causal reasoning refers to the process or set of processes by which we arrive at judgments about cause and effect in a wide range of situations. The process by which we acquire the knowledge on which causal reasoning is based is referred to as causal learning. Since at least the British empiricists of the eighteenth century (Hume, 1978), causal reasoning has been linked to mental (and statistical) association. The study of human associative learning concerns how we acquire information about covarying events in our environment and the inferences and decisions that we make as a consequence of experiencing those events. As a field, its aims overlap with those of causal reasoning, though they are distinct in important ways. For instance, human associative learning research has typically been more concerned with how we acquire information about the covariation of events, than how this information is translated into judgments about cause and effect. The assumption generally taken is that (other factors being equal) the stronger the association between a cue and outcome, the stronger the causal judgment (Thorwart and Livesey, 2016; Le Pelley et al., 2017). The notion that we often interpret associations between events as evidence of a causal relationship has stimulated plenty of debate about the nature of causal learning as well as the nature of associative learning in humans in other domains (e.g., Mitchell et al., 2009). Formal models of associative learning make predictions about how covariation between events leads to differences in the strength of associations (e.g., Rescorla and Wagner, 1972) or the strength of retrieved memories (e.g., Stout and Miller, 2007). Many studies have shown that people make causal judgments that are sensitive to factors, which are not captured by these models (e.g., see Shanks, 2007; Mitchell et al., 2009 for reviews). In particular, instructions that manipulate the relevance of various properties of the learning context can influence whether causal judgments conform to the predictions of associative learning models. For instance, prior understanding of how causal relationships work within a given domain can determine whether and to what extent causal judgments exhibit competition among simultaneously presented cues (Waldmann and Holyoak, 1992; Waldmann, 2001). This study examined a related but distinct question, whether instructions to ignore or to use prior knowledge can control competition for associative memory in the same way that they control cue competition in causal judgments.

Cues that occur together interact lawfully in ways that suggest competition for a limited amount of learning that an outcome can support. For instance, two novel cues presented in compound and followed by an outcome (AX+) appear to support less learning than when each cue is paired with the outcome separately (A+/X+). When A is more salient than X, learning about the relationship between A and the outcome appears to heavily “overshadow” learning about X (Mackintosh, 1975). However, when the cues are of approximately equal salience (as is often the case in human learning experiments), this competition appears to be reciprocal such that neither cue achieves the level of association that it would if trained in isolation (Mackintosh, 1976 or see, e.g., Mitchell et al., 2006, for evidence of this in human causal learning). This mutual competition between cues is widely referred to as overshadowing (e.g., McLaren et al., 2014). Another prominent example of cue competition is the blocking effect. In the typical procedure one cue, B, is reliably followed by an outcome in an initial stage (B+). Later, this cue is presented in compound with a novel cue (Y) and again is followed by the outcome (BY+). In this instance, responding to the target cue Y is often observed to be weaker than responding to control cues A or X, suggesting that learning about the target cue Y is blocked by previous learning that B reliably predicts the outcome (Kamin, 1968). Cue competition effects were initially discovered in animal conditioning; however, there is now a wealth of evidence to suggest that they also occur in human causal learning (e.g., Shanks, 1985; Aitken et al., 2000; Livesey et al., 2013; Le Pelley et al., 2017).

Cue competition effects like blocking and overshadowing are consistent with the idea that the strength of learning is determined by prediction error or the discrepancy between the expectancy of an outcome and what is actually experienced (Rescorla and Wagner, 1972). Prediction error models of learning that assume a summed error term (in which the expectancy of an outcome is a product of the summed associative strength of all cues present on a trial) have been highly influential in the development of associative learning theory and its application to human causal learning (Shanks, 2007). By such accounts, blocking occurs because the pretraining of cue B leads to a strong expectation of the outcome on BY+ trials. When the outcome occurs (+), prediction error is minimal and therefore Y does not develop a strong association with the outcome (Rescorla and Wagner, 1972). Other associative models assume that attentional processes are implicated in the blocking effect (e.g., Mackintosh, 1975; Pearce and Hall, 1980). For instance, Mackintosh (1975) proposed that the predictive validity of a cue determines its associability and thereby the attention paid to it. Thus, pretraining renders B more salient in the context of the task and blocking is at least partly the result of a lack of attention to the blocked cue. Consistent with this account, a blocking procedure does appear to produce biases in attention away from the blocked cue in human causal learning tasks (e.g., Beesley and Le Pelley, 2011). Reduced prediction error should limit learning about those specific aspects of an outcome that are predicted. Predictions that are based on prior learning should not restrict further learning about an unexpected outcome or an unexpected property of the outcome. It should be noted that individual models like Rescorla-Wagner and Mackintosh are unable to provide a complete account of associative learning, but as Le Pelley et al. (2017) recently argued, the failings of any one these models in the context of human causal learning does not undermine the evidence that the general principles of associative learning (in particular, the assumption that prediction error determines the strength of learning) apply to human learning regardless of whether the relationships under question are causal or non-causal.

When studying human causal learning in the laboratory, researchers often use hypothetical scenarios to establish a framework for learning novel relationships. In many such studies, researchers capitalize on existing knowledge by selecting scenarios that are intuitive and familiar. Participants are asked to disregard their prior knowledge when making judgments in the task, but the assumption made by researchers is that new learning will proceed quickly because the cues are easy to identify and discriminate. One widespread example is the allergist task (Wasserman, 1990). In this task, participants assume the role of an allergist who is trying to identify the specific allergies of a fictitious patient (Mr. X). On each trial, a meal (consisting of one or more foods) that Mr. X has eaten is presented, and participants are asked to predict whether or not Mr. X will suffer an allergic reaction. After each prediction, they receive corrective feedback. Finally, participants are asked to judge which foods are causing Mr. X to suffer an allergic reaction, and this judgment may also take the form of a probability or cued recall judgment. The food allergist task has been used to study cue competition effects such as blocking and overshadowing (e.g., Shanks and Lopez, 1996; Aitken et al., 2000; Lovibond et al., 2003; Beckers et al., 2005a; Mitchell et al., 2005, 2006; Vandorpe et al., 2007; Livesey et al., 2013, 2019b; Luque et al., 2013; Uengoer et al., 2013), learning of preventative relationships such as in the case of conditioned inhibition (Karazinov and Boakes, 2004, 2007), complex rule learning tasks such as the patterning task (Shanks and Darby, 1998; Wills et al., 2011; Don et al., 2020), as well as a host of phenomena related to learned attentional changes including the learned predictiveness effect (Le Pelley and McLaren, 2003; Don and Livesey, 2015; Shone et al., 2015), the inverse base-rate effect (Don et al., 2019), outcome predictability effects (Griffiths et al., 2015; Thorwart et al., 2017), and other related transfer effects (Livesey et al., 2019a). Food allergies are relatively commonplace such that, by the time they enter the laboratory, participants have a lifetime of experience with food and its ability to cause allergic reactions in oneself or others. These properties not only support learning new relationships established in the experiment but also mean participants bring to the experiment prior knowledge or biases that may not be easily set aside.

In this study, we examined the extent to which participants are able to suppress prior knowledge and beliefs, when instructed to do so, in a causal learning task. We tested the extent to which prevailing cue-outcome associations (i.e., associations that people typically hold about certain foods and types of allergies) influenced cue competition expressed in both causal ratings and in the strength of associative memory. We first surveyed an independent sample to identify a number of foods commonly (and uncommonly) associated with allergic reactions. We then used this information to create a pseudo-blocking design in which the pretraining was replaced by prevailing beliefs about food allergies. That is, the foods rated as highly allergenic and those given a low allergenic rating were paired with foods that given a moderate allergenic rating to create two compounds; high + moderate and low + moderate. Each type of compound was then associated with the allergic reaction outcomes. Participants were either told to use or ignore their prior knowledge of foods and allergies. For participants told to use their knowledge, we assumed that the presence of a high allergenic food in high + moderate compounds would generate a prediction that an allergic reaction was going to occur, and that when it did, participants would attribute this outcome to the high allergenic food more than the moderate food. We expected to see evidence of this in both causal ratings and associative retrieval such that, for this “use your prior knowledge” group, moderate foods paired with low-allergenic foods would have higher causal ratings and higher associative memory scores than moderate foods paired with high allergenic foods. The key question was to what extent this pattern changed when participants were told to *ignore* their prior knowledge. In other words, do people have control over whether prior beliefs influence their current judgments and is that control the same for causal and associative memory judgments? As mentioned, past studies have shown that causal judgments are sensitive to instructions that inform how covariation information applies in a given context (Waldmann and Holyoak, 1992; Waldmann, 2001). Thorwart and Livesey (2016) argued that such instructions could also affect how that covariation information is acquired in the first place.

In order to achieve this end, we conducted an initial survey of a separate sample of undergraduate psychology students from the University of Sydney. The survey included 30 common food items and participants were asked to indicate the extent to which they associated each food with an allergy as well as which specific allergenic symptoms they associated with allergies to the foods in question. From the survey, we were able to identify three categories of foods, those strongly associated with allergy, those weakly associated with allergy, and some moderately associated with allergy.

If prevailing beliefs about food allergies lead participants to generate predictions about the likelihood of an allergic reaction, then prediction error models of learning would predict that compounds with high allergenic foods should generate less prediction error than compounds with low allergenic foods. Given the evidence outlined above, we therefore expected that food cues commonly associated with allergy would produce greater competition for association with the outcome than foods infrequently associated with allergy, thereby leading to poorer learning for the moderate cue-outcome relationship in the high + moderate than the low + moderate compounds.

The highly allergenic foods identified in the survey formed two subcategories based on the kind of symptoms most strongly associated with them; the first were associated with anaphylactic type symptoms (for example, difficulty breathing, swelling, and rash) and the second, gastrointestinal symptoms (such as stomach ache, cramps, and nausea). These two subcategories enabled us to manipulate the specific properties of the outcome of high + moderate compounds. Specifically, the experienced allergic reaction could be either consistent or inconsistent with the category of symptoms commonly associated with the high allergenic food in question. This is important because the highly allergenic foods may increase competition for learning because they reduce prediction error or they may simply increase competition because they are more salient in the context of the allergist task. That is, highly allergenic foods could strongly overshadow their moderate competitors as their prior predictive validity in such scenarios would lead to an attentional bias toward them in the manner proposed by Mackintosh (1975). If so, then the relative consistency of the type of allergy that follows should not affect the strength of learning, and we should see no difference in associative memory among the high + moderate compounds. On the other hand, experiencing an allergic reaction that is inconsistent with expectations ought to generate a larger prediction error than one that is consistent. Therefore, we may see greater competition produced by an outcome that is consistent than one that is inconsistent with expectations. Prediction error driven learning would therefore predict greater overshadowing when the experienced symptom of the allergic reaction is commonly associated with the competing cue than when it is not.

Using the food cue and allergic reaction outcome categories identified from this survey, we constructed the design shown in Table 1. As noted earlier, two kinds of compound formed the basis of the pseudo-blocking cue competition design: high + moderate and low + moderate. The high + moderate compounds were paired with a specific reaction that was either consistent or inconsistent with expectancies. For example, a food commonly associated with gastrointestinal symptoms, milk, for example, could be paired with a reaction that was either consistent with this pattern (e.g., stomach ache) or inconsistent with this pattern (e.g., rash). Half of the sample was given the typical instruction to ignore what they know about food allergies in the real world (group *ignore*), and the remainder were told that their existing knowledge of food allergies would be useful and that they should use that knowledge to inform their judgments (group *use*). In other words, the instructions were intended to encourage or discourage participants from relying on their prior knowledge of food allergies when making predictions in the task. If participants are able to set aside their existing beliefs when making judgments about the causal relationships in the task, then those who are instructed to use their prior knowledge of food allergies should show greater competition, and therefore, a stronger distinction in ratings for the moderate cues paired with high vs. low allergenic foods, than those who are told that their prior knowledge is not informative. Given the evidence outlined above, we expected causal ratings to be sensitive to instructions to use or ignore prior knowledge. The question was whether associative memory judgments would reflect the same level of control.

**Table 1:** Experiment design.

Compounds	Training	Test
High + Moderate	**A**R – O1_con_	**A**, R
	**B**S – O2_inc_	**B**_,_ S
	**C**T – no O	**C**_,_ T
	**D**U – O3_con_	**D**, U
	**E**V – O4_inc_	**E**, V
	**F**W – no O	**F**, W
		
Low + Moderate	*H*X – O5	*H*, X
	*I*Y – O6	*I*_,_ Y
	*J*Z – no O	*J*_,_ Z

Letters represent food cues randomly assigned within subcategories: Those in **bold** (A–F), those in *italics* (H–J), and those in regular font (R–Z) are foods identified as strongly associated with allergies, weakly associated with allergies, and moderately associated with allergies, respectively. Outcomes 1–6 fall into two subcategories, stomach-related (nausea, cramps, and stomach ache) and anaphylaxis-related reactions (difficulty breathing, rash, and swelling). Cues A–C are those foods generally associated with anaphylactic symptoms and D–F gastrointestinal symptoms. Highly allergenic foods were paired with an outcome either consistent or inconsistent with expectations, e.g., if A were *peanuts* and B were *almonds* (two foods associated with anaphylactic symptoms), then O1 would be *difficulty breathing* and O2 would be *stomach ache*. This relationship is denoted by the subscript on the outcome: “con” for consistent and “inc” for inconsistent.

## Materials and Methods

### Participants

We recruited 137 undergraduate psychology students from the University of Sydney to participate in the experiment. Sixty-six of these students completed the experiment as part of a tutorial assessment for an advanced psychology course in learning and behavior, and the remainder were recruited from the first year psychology subject pool and received partial course credit as compensation for their participation. Of these, two were excluded on the basis of failing to meet the learning criterion (set at <50% accuracy across training) and further 11 were excluded for failing the manipulation check (described below). The remaining sample of 124 had a mean age of 21.02 years and was predominantly female (*N* = 85). Half were randomly assigned to the *ignore* condition (*n* = 62) and half to the *use* condition (*n* = 62).

### Stimuli

An independent sample of 74 undergraduate psychology students from the University of Sydney were asked to complete a short survey of their knowledge or intuitions of common food allergies in exchange for course credit. Of these, six were excluded for non-compliance, and leaving a final sample of 68 participants. Participants were shown 30 common food items and were asked to complete two questions about each food in turn. The first was to rate the extent to which each item was associated with an allergy of any kind on a scale ranging from “not at all” to “strongly associated.” They were then asked to identify any specific symptoms associated with an allergic reaction to the foods in question, for example, difficulty breathing. Participants were instructed to select one or more symptoms from a list (which included “NA” as an option for those foods that were not associated with an allergy or for which there was no specific symptom that they associated with that food allergy) and were given the option to specify any that were not listed. Eighteen foods were selected for this experiment, six from among the highest mean association with allergy, three of the lowest mean association with allergy, and the remaining nine from the cues that fell in the moderate range between these two extremes. The results of this survey are provided in the Supplementary Material.

### Procedure

The allergist task was programed with the Psychophysics Toolbox for MATLAB (Kleiner et al., 2007). Participants were given a hypothetical scenario in which they were asked to play the role of an allergist trying to determine the allergies of a particular patient, Mr. X. Half of the participants were given the standard instructions that usually accompany such tasks, that is, they were told to ignore what they know about food allergies and use only the information presented to make their judgments. The remaining participants were told that the patient’s allergies were based on real world examples, and thus they should assume that any knowledge of food allergies that they possess would be useful in making judgments about this patient’s allergies. Training consisted of four blocks of 18 trials in which each compound in Table 1 was presented twice per block. On each training trial, participants were presented with a meal consisting of two foods that Mr. X had eaten and were asked to predict which allergic reaction (if any) would occur as a result. They used the mouse to select an outcome from among seven possible options (*nausea*, *cramps, stomach ache*, *difficulty breathing*, *rash*, *swelling*, or *no allergic reaction*). After a choice was made, corrective feedback was presented onscreen for 2 s before the next trial began. In order to reduce recency effects in the memory test, participants completed a short filler task after the training phase. The filler task was a set of eight syllogisms adapted from the belief bias task (Markovits and Nantel, 1989). Each item presented a conclusion that was either believable but invalid or unbelievable but valid. The syllogisms were presented one at a time, and participants were asked to judge if each conclusion was logically true or false. The results of the filler task are reported in the Supplementary Material.

At test, participants were presented with the cues from the training phase one at a time and were asked to make two different judgments about each cue. First, they were asked to recall the outcome that had followed the cue in question. All seven possible outcomes were presented onscreen and participants made a selection by clicking on the corresponding label. Once they had done so, they rated their confidence in their choice by indicating on a linear analogue scale ranging from “not at all confident” to “very confident.” Second, they were asked to make a causal judgment about the cue in question. Another linear analogue rating scale appeared onscreen and participants were asked to indicate to what extent they believed the cue caused an allergic reaction of *any* kind in the fictitious patient Mr. X. Following this, a manipulation check was administered in which participants were asked to indicate which version of the critical instructions they had received at the beginning of the experiment. Three options were presented on screen, each of the critical instructions in full or “neither of the above.” Twelve participants who failed to correctly report the instruction they received were excluded from the analysis, seven of these were from the ignore group and the remaining five from the use group.

Finally, to gauge the extent to which our participant’s prior beliefs about food allergies aligned with those of the independent sample used to inform this design, participants completed a questionnaire about their prior knowledge or understanding of the allergenic properties of the foods used as cues in the experiment. They were instructed to ignore the events of the experiment when answering the questionnaire and respond based on their knowledge about these foods *before* the experiment began. Of course, giving such instructions presupposes that participants can successfully set aside what they experience in the experiment when responding on this questionnaire, the very question we aimed to address in this study. To anticipate the results, we found that prior knowledge biased associative retrieval even when participants were asked to ignore that knowledge. This suggests that any consistency between the initial survey and responses on the post-experimental questionnaire should be interpreted with caution. The questionnaire was programed and conducted using the Qualtrics platform and was identical to the initial survey discussed above, with the exception that only the 18 food items retained for the experiment were included.

## Results

Bayesian analyses were conducted with the “BayesFactor” package (Morey and Rouder, 2018) for R (R Core Team, 2019).

### Training

**Figure 1:**
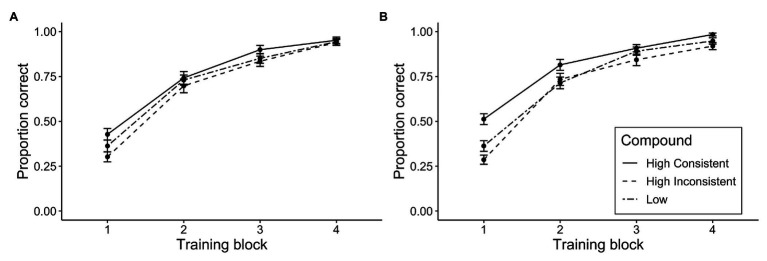
Mean proportion correct during training for **(A)** the ignore group and **(B)** the use group as a function of compound and training block. Compound names refer to the status of the competing cue. For example, “High Consistent” summarizes accuracy data for compounds with one highly allergenic cue that were followed by a symptom consistent with expectancies derived from the initial survey (AR and DU from Table 1). For simplicity, we have not included data from the filler compounds paired with “no allergic reaction,” but we report this training data in the Supplementary Material. Error bars represent standard error of the mean.

Figure 1 shows the mean proportion correct predictions in the training phase as a function of cue type and instruction condition. Among participants that met the training criteria, mean accuracy in the final block of training was above 90% in both instruction groups, indicating that both groups were able to learn the contingencies. To investigate whether there were any differences between groups in the strength of acquisition of the compounds of interest, we conducted a three-way ANOVA on training accuracy with factors of compound (high consistent + moderate vs. high inconsistent + moderate vs. low + moderate), training block (1–4), and instruction group (ignore vs. use). As a complement to this analysis, we conducted an equivalent Bayesian repeated measures ANOVA with default priors for the effects (*r* = 0.5). For the interaction, we report the Bayes factor inclusion across matched models, which provide an estimate of evidence for the effect by comparing the model with the interaction effect against equivalent models stripped of the effect (denoted BF_Inclusion_; Rouder et al., 2017). There was a significant effect of block, *F*(2.56, 312.84)^1^ = 627.58, *p* < 0.001, *η_p_*^2^ = 0.846, *BF_10_* = 1.33 × 10^259^, indicating that performance improved with training. There was also however a significant main effect of compound, suggesting that there were some differences in accuracy for the different compounds, *F*(1.98, 241.13) = 16.10, *p* < 0.001, *η_p_*^2^ = 0.117, *BF_10_* = 252. However, as the interaction with block implies, these differences were significantly diminished by the end of training, *F*(5.06, 617.66) = 4.46, *p* < 0.001, *η_p_*^2^ = 0.035, *BF_Inclusion_* = 2.47. Neither the main effect of instruction nor any other interactions reached significance, the largest *F* = 1.47 for the compound by instruction interaction.

### Test

#### Learning Scores

**Figure 2:**
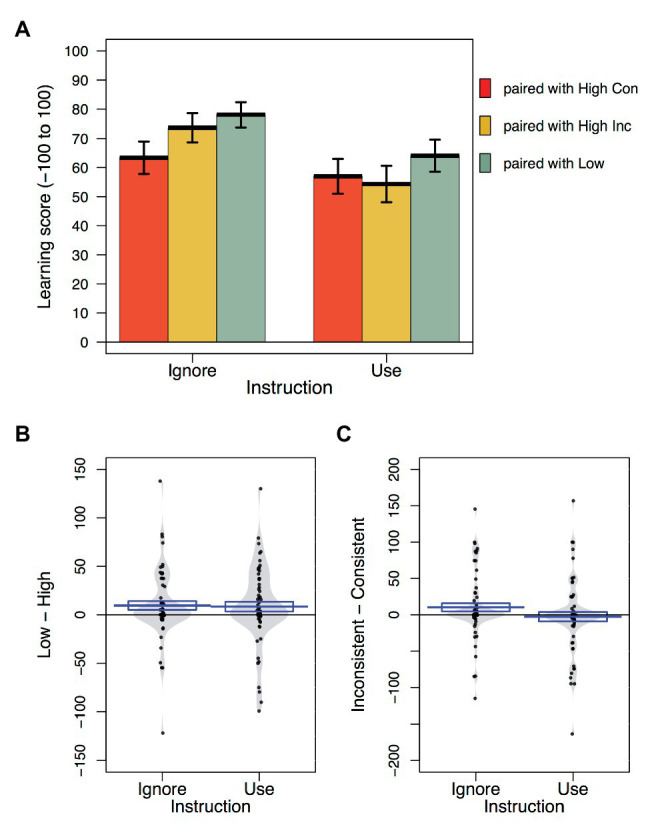
Learning scores as a function of cue type and instruction group. **(A)** Mean, and standard error of learning scores for each cue type as a function of instruction group. **(B)** Violin plots showing the distribution of learning score differences between moderate cues paired with low and high allergenic cues. **(C)** Violin plots showing the distribution of difference scores between cues paired with high consistent and high inconsistent foods. Box around mean line represents standard error of the mean.

Choice accuracy and confidence ratings from the memory test were converted to a learning score reflecting the strength of associative recall for each cue. Learning scores were calculated by multiplying choice accuracy (coded 1 for a correct response and −1 for an incorrect response) by confidence rating (ranging from 0 to 100). Thus learning scores could range from −100, indicating strong memory for an outcome that was not paired with the cue during training, to 100, reflecting strong recall of the correct outcome. Of primary interest were the learning scores for the moderate cues that were presented with either a high or low allergenic food during training. Figure 2A shows the individual and mean learning scores for the moderate cues (R/U/S/V/X/Y) as a function of instruction group. Learning scores were analyzed by means of a set of two planned orthogonal contrasts that answered our specific hypotheses. The first compared the scores for moderate cues paired with high allergenic foods (regardless of the consistency of outcome) with scores for moderate cues paired with low allergenic foods (high vs. low).^2^ The second, compared scores for moderate cues paired with high consistent with scores for moderate cues paired with high inconsistent cues (consistent vs. inconsistent). These comparisons are represented in Figures 2B,C, which show individual and mean difference scores for each contrast (low – high and inconsistent – consistent, respectively) as a function of instruction group. These contrasts were complemented with Bayesian *t*-tests, comparing the evidence for the comparisons against a null hypothesis. For each of these tests, we specified a non-directional alternative assigned a Cauchy distribution with default scaling *r* = 0.707.

Learning scores were on average lower for moderate cues paired with high allergenic foods (R/U/S/V) than for moderate cues paired with low allergenic foods (X/Y), *t*(122) = 2.63, *p* = 0.010, *η_p_* ^2^ = 0.054, *BF_10_* = 2.78. This pseudo-blocking effect in associative memory did not appear to differ in magnitude across instruction groups, *t*(122) = 0.17, *p* = 0.865, *η_p_* ^2^ < 0.001, *BF_01_* = 5.15 in favor of the null. For moderate cues paired with high allergenic foods, there was no significant difference in learning scores for those followed by consistent (R/U) vs. inconsistent outcomes (S/V), *t*(122) = 0.78, *p* = 0.379, *η_p_* ^2^ = 0.006, *BF_01_* = 6.88 in favor of the null, and no significant interaction with group, *t*(122) = 0.92, *p* = 0.136, *η_p_* ^2^ = 0.018, *BF_01_* = 1.89 in favor of the null.

For completeness, we also analyzed the scores and ratings for the pseudo-blocking cues A–F; however, we note that the interpretation of these analyses is complicated for two reasons. First, it was not possible to randomly allocate the foods to high or low allergenic categories as it was for the moderate cues so this comparison is not properly counterbalanced. Secondly, these ratings do not inform our hypotheses. That is, whether or not we can ignore prior beliefs about food allergies when making judgments about them is a different question to whether these beliefs have an impact on learning about other cues with which they are competing. Associative memory for the pseudo-blocking cues A–F that had either a strong or weak prior association with allergy was overall quite strong. We ran the same set of contrasts for the pseudo-blocking cues, as for the moderate cues of interest. On average, learning scores for highly allergenic foods (A/B/C/D; *M* = 63.30, *SE* = 3.34) did not differ significantly from those for low allergenic foods (E/F; *M* = 62.49, *SE* = 4.23), *t*(122) = 0.190, *p* = 0.849, *η_p_* ^2^ < 0.001, *BF_01_* = 9.85 in favor of the null. This was true regardless of whether or not participants were instructed to use or ignore this information as there was no significant interaction with instruction group, *t*(122) = 1.15, *p* = 0.252, *η_p_* ^2^ = 0.011, *BF_01_* = 2.89 (mean difference high – low was −4.09 (*SE* = 5.79) in the ignore group and 5.71 (*SE* = 6.23) in the use group). Similarly, there was no main effect of consistency on memory for the high allergenic foods, *t*(122) = 0.17, *p* = 0.865, *η_p_* ^2^ < 0.001, *BF_01_* = 2.59 (high consistent *M* = 67.76, *SE* = 4.09; high inconsistent *M* = 58.85, *SE* = 4.44) and no significant interaction with instruction group, *t*(122) = 1.51, *p* = 0.135, *η_p_* ^2^ = 0.018, *BF_01_* = 1.88 (the mean difference consistent – inconsistent was 0.94 (*SE* = 6.99) and 16.89 (*SE* = 7.96) for the ignore and use groups, respectively).

#### Causal Ratings

**Figure 3:**
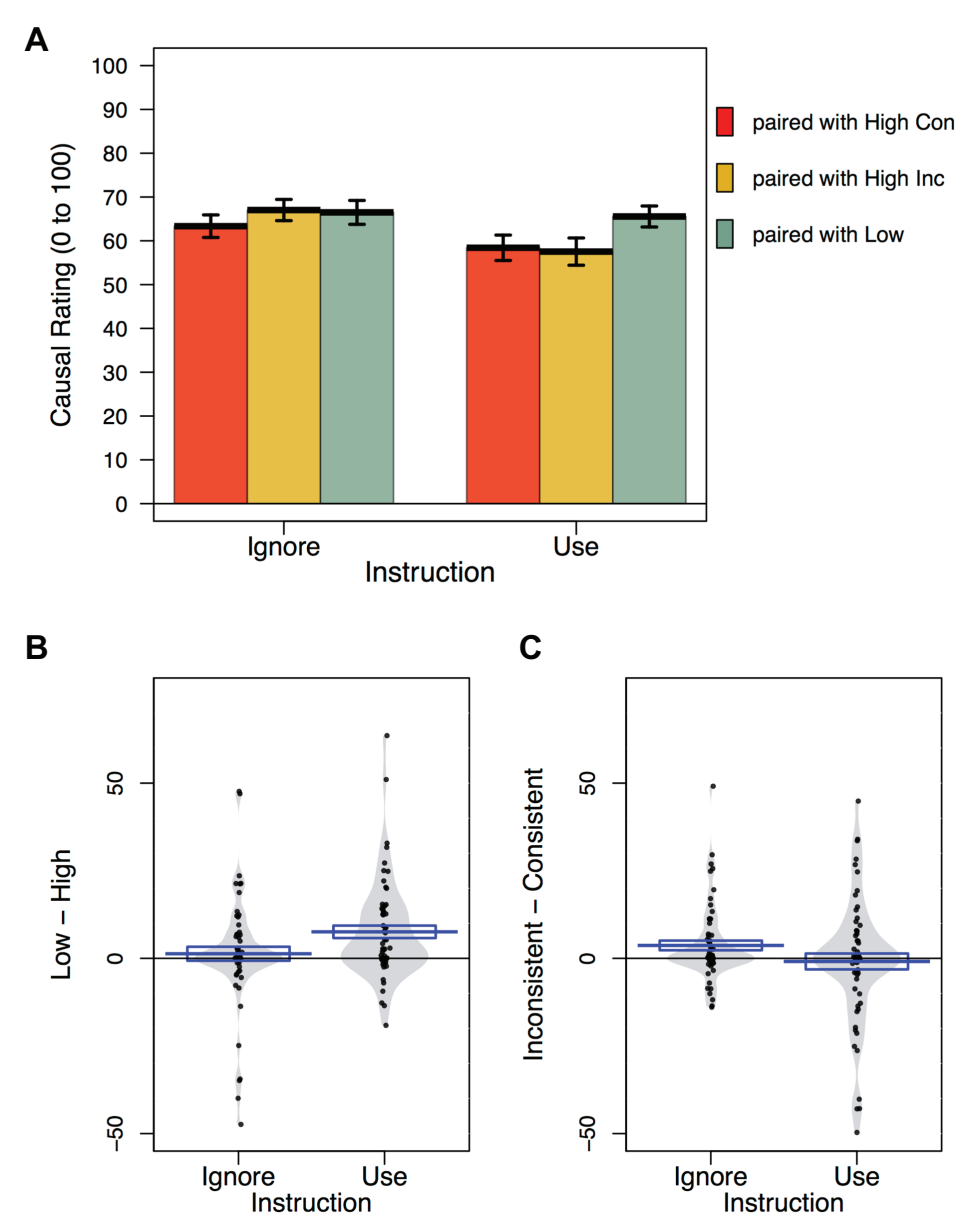
Causal ratings as a function of cue type and instruction. **(A)** Mean, and standard error of causal ratings for each cue type as a function of instruction group. **(B)** Violin plots showing the distribution of causal rating differences between moderate cues paired with low and high allergenic cues for each instruction group. **(C)** Violin plots showing the distribution of differences between causal ratings for cues paired with high consistent and high inconsistent foods. The box around mean line represents the standard error of the mean.

Causal ratings for the critical cues are illustrated in Figure 3. Causal ratings were analyzed in the same way as learning scores. Consistent with the learning scores, there was evidence of cue competition with causal ratings for the moderate cues paired with low allergenic foods being significantly higher on average than those paired with high allergenic foods (high vs. low), *t*(122) = 3.33, *p* = 0.001, *η_p_* ^2^ = 0.083, *BF_10_* = 15. However, the magnitude of pseudo-blocking was significantly larger following instructions to use prior knowledge than to ignore it, *t*(122) = 2.35, *p* = 0.021, *η_p_* ^2^ = 0.043, *BF_10_* = 2.25. Bayesian *t*-tests for each instruction group confirmed that participants instructed to use their prior knowledge gave significantly higher causal ratings to moderate cues that were paired with low allergenic foods (X/Y) than with high allergenic foods (R/U/S/V), *BF_10_* = 285, *t*(61) = 4.25, *p* < 0.001, *d* = 0.54. Whereas those instructed to ignore their prior knowledge causal ratings for cues paired with high allergenic foods did not differ significantly from those for cues paired with low allergenic foods, *BF_01_* = 5.843 in favor of the null, *t*(61) = 0.66, *p* = 0.513, *d* = 0.084. The second contrast, comparing ratings for moderate cues paired with high consistent with high inconsistent competitors did not reach significance, *t*(122) = 1.06, *p* = 0.291, *η_p_* ^2^ = 0.009, *BF_01_* = 5.84 in favor of the null, nor was there a significant interaction with group, *t*(122) = 1.72, *p* = 0.088, *η_p_* ^2^ = 0.024, *BF_01_* = 1.38 in favor of the null.

For the pseudo-blocking cues A–F, we found that the high allergenic foods (A/B/C/D; *M* = 69.18, *SE* = 1.59) were on average given significantly higher causal ratings than the low allergenic foods (E/F; *M* = 61.84, *SE* = 1.89), *t*(122) = 4.23, *p* < 0.001, *η_p_* ^2^ = 0.128, *BF_10_* = 337. However, there was no significant interaction with instruction group (*t*(122) = 1.46, *p* = 0.146, *η_p_* ^2^ = 0.017, *BF_01_* = 1.99), indicating that even when participants were instructed to ignore their prior knowledge low allergenic cues were considered to be less likely to be causing an allergic reaction than the high allergenic cues [*MD* (high – low) = 4.80; *SE* = 1.71 in group ignore; *MD* = 9.88, *SE* = 3.02 in group use]. Comparing the high allergenic cues on consistency of outcome, high consistent cues (A/C; *M* = 60.87, *SE* = 1.95) were given higher mean causal ratings than high inconsistent cues (B/D; *M* = 62.28, *SE* = 2.01), *t*(122) = 3.01, *p* = 0.003, *η_p_* ^2^ = 0.069, *BF_10_* = 7.37. This was not affected by the critical instructions, *t*(122) = 0.79, *p* = 0.433, *η_p_* ^2^ = 0.005, *BF_01_* = 3.94 [*MD* (consistent – inconsistent) = 3.15, *SE* = 1.61 in group ignore, and *MD* = 5.38, *SE* = 2.33 in group use].

### Post-experimental Questionnaire

**Figure 4:**
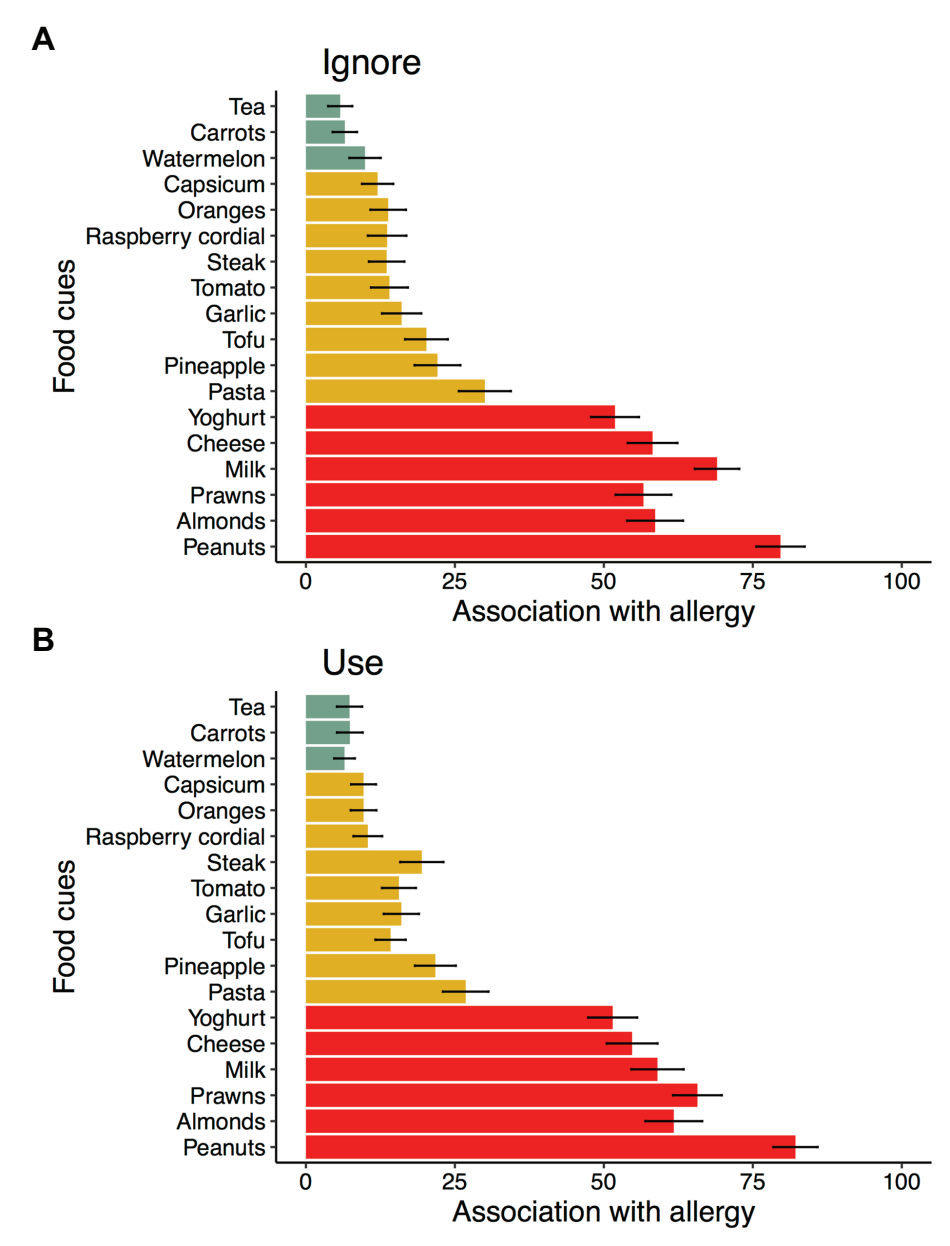
Mean ratings of association with allergy from the post-experiment questionnaire for those instructed to ignore **(A)** or use **(B)** their knowledge of food allergies during the allergist task. Colors represent the categories low (green), moderate (yellow), and high (red) allergenic as identified from the previous survey of a separate sample of participants. Error bars represent standard error of the mean.

Results from the post-experimental questionnaire assessing the strength of association with allergy for each food at the beginning of the experiment were consistent with those drawn from the survey used to create the design. Figure 4 shows the mean association with allergy for each food item in the experiment as a function of instruction group. The ratings for each cue were collapsed across categories identified in the initial survey (high, moderate, and low) and subjected to a cue category by instruction group ANOVA. There was a significant main effect of cue category, *F*(1,366) = 366.57, *p* < 0.001, that did not interact with instruction group, *F* < 1.

## Discussion

This study tested the assumption that participants can successfully follow instructions to set aside existing beliefs or knowledge about causal relationships and learn new cue-outcome relationships in an unbiased way. Using a cue competition design in the allergist task, we demonstrated a pseudo-blocking effect in both associative recall and causal judgments. That is, foods commonly held to be highly associated with allergies produced less prediction error and, therefore, greater overshadowing, than foods rarely associated with allergies. Critically however, causal judgments reflected some level of instructed control over this process. Those explicitly instructed to ignore rather than use their prior knowledge of food allergies when completing the task showed no evidence of differential competition for high and low allergenic foods in their judgments of causality. This was not true however of associative recall, high allergenic cues produced greater overshadowing than low allergenic cues regardless of whether or not they were instructed to take their prior knowledge into consideration when completing the task.

It is not uncommon in causal learning research to manipulate instructions in an attempt to control the relevance of previous information to the current situation. Sometimes this takes the form of a standalone manipulation aimed at encouraging participants to attend or attribute causation to cues in a different way to what they have learned previously (e.g., Mitchell and Lovibond, 2002; Mitchell et al., 2012; Don and Livesey, 2015; Shone et al., 2015; López et al., 2016). In other experiments, such instructions complement demonstrations made *via* cue-outcome pre-training trials (e.g., Lovibond et al., 2003; Livesey and Boakes, 2004; Beckers et al., 2005a). Many of these results have shown that people’s causal judgments are shifted in rational ways by such instructions. Here, we demonstrated the same general sensitivity, at least for causal ratings. When judging the ambiguous causal connections between food cues and allergic reaction outcomes, people told to ignore what they knew about food allergies treated the cues in this task as if they were all equally likely to be allergenic. However, the results also indicate that the strength of associative memory may be less affected by such instructions.

What does this mean? Thorwart and Livesey (2016) suggested that even if it was assumed that prediction-error driven association formation comprised a core memory system on which causal inferences might be based then there were at least three ways in which other types of knowledge (for instance, inferences and assumptions drawn from instructions) might impact on the learning that takes place within that associative system. Perhaps the simplest of these would be to assume that the instructions modify the way associative memories are translated to explicit decisions and judgments, without strongly influencing the way those memories are laid down in the first place. In other words, we could assume that the instruction to ignore prior knowledge leads participants to give a similar causal rating to all ambiguous cues but has little impact on the way participants learn about them. It should be noted that this is a possible explanation for many of the other demonstrations of instruction-based manipulations of learning.

If this were the case then we could hypothesize which aspects of memory encoding are resistant to being modified by relevant instructions, like the instruction to ignore prior knowledge. One possibility is that the encoding of the cues, their initial sampling and the distribution of selective attention are unaffected by these instructions. That is, highly allergenic foods might attract more attention than less allergenic foods, regardless of whether participants are told to use or ignore their prior knowledge. Another possibility is that predictions made during memory encoding (i.e., those relevant to prediction-error based learning) are resistant to this instruction manipulation. Predictions about allergic reaction outcomes might be automatically retrieved based on prior memories in a way that impacts competitive learning even when the participant has been told to ignore this information.

The lack of any strong difference between the consistent and inconsistent cue-outcome pairing conditions is more consistent with the first of these possibilities. In the inconsistent condition, prior knowledge of the outcomes typically associated with highly allergenic foods would lead to the prediction of an incorrect outcome based on the highly allergenic food in the compound. Although the participant would be correct in anticipating an allergic outcome of any type, there would presumably still be greater prediction error in this condition. According to associative learning algorithms that assume a summed error term, this should drive stronger learning about the moderate cue presented in this compound. In contrast, if there were a persistent bias toward the highly allergenic cue during learning then this might impact learning of the moderate cue regardless of whether the outcome presented was consistent or inconsistent with the participant’s prior beliefs. We tentatively suggest then that the results reflect a persistent bias in cue encoding in particular, though we cannot rule out the possibility that the instructions failed to influence other aspects of learning and memory also.

A selection bias toward the highly allergenic cues is consistent with an attentional account of cue competition effects like blocking (Mackintosh, 1975). There is empirical support this explanation of blocking, as blocked cues are slower to enter into new learning, consistent with a decrease in associability (Kruschke and Blair, 2000; Le Pelley et al., 2007). Further, there is evidence that these changes occur very rapidly. Luque et al. (2018) used a dot probe task to show that a blocking procedure produces very early shifts in attention away from the blocked cue consistent with a learned reduction in the perceived salience of the blocked cue. While we do not measure attention directly here, our data are certainly compatible with such an account if prior knowledge can increase cue salience in a similar manner to pretraining. A corollary of the idea that cue competition entails very rapid and perhaps automatic changes in attention is that these selection biases are likely to be somewhat resistant to control by instructions. While there is some evidence that learned attentional biases are under voluntary control as they can be completely reversed by instructions alone (Mitchell et al., 2012), partial resistance to instructed changes in attention have been documented in other contexts involving causal learning (e.g., Don and Livesey, 2015; Shone et al., 2015). Perhaps the most convincing evidence of such resistance comes from a recent study by Cobos et al. (2018), in which they demonstrated that the selection history of a cue, its previous predictive value, produces a very rapid attentional shift that is resistant to instructed control. However, it should be noted that even in these studies there was still substantial evidence that instructions modified both causal ratings and associative memory ratings.

The observed dissociation between associative memory and causal judgments raises another question about the relationship between these judgments. As noted earlier, there are clear parallels between associative and causal learning, both are subject to cue competition, for instance, that have led some to conclude that the same processes inform both learning and causal judgments. However, it is clear that associative models cannot provide a full account of human causal judgments and the evidence here is consistent with previous findings that causal judgments are sensitive to factors that fall outside the scope of associative models of learning (Waldmann and Holyoak, 1992; Waldmann, 2001; Beckers et al., 2005b). However, this does not preclude the notion that the strength of associative memory may serve as a useful heuristic for establishing causality in the absence of other information (Thorwart and Livesey, 2016; Le Pelley et al., 2017).

Cues were always trained in a compound of two cues such that, in the absence of any prior knowledge about the allergenic properties of cues, the causal status of *all* cues (aside from those never paired with an allergic reaction) was ambiguous. Thus, even if associative memory was weaker for those moderate cues paired with highly allergenic competitors, in reflecting upon the instruction to ignore their prior knowledge, participants could infer that all cues have the same relationship with the outcome and adjust their causal ratings accordingly. That is, causal ratings could be based on the strength of associative recall, as they appeared to be when participants were told to use prior knowledge, but participants may also be able to reflect upon the validity of this evidence when making these judgments at test. It appears that prior knowledge of causal or predictive relationships, regardless of how it is acquired, influences competition for associative memory and that this process is resistant to control by instructions. The question is whether this is also true of previous learning. In other words, are direct experiences of cue-outcome relationships gained within the same experimental context similarly resistant to instructed control, and if so, does this affect associative memory and causal judgments equally? Future research could address this question by using a combination of pretraining and instructions.

In summary, we have demonstrated that associative memory is relatively insensitive to instructions manipulating the relevance of prior knowledge. We observed that while participants could successfully set aside their prior knowledge when making causal judgments about the relationships in the task, instructions to ignore prior knowledge did not affect competition for associative memory within the task. That is, prevailing beliefs about food allergies affected learning, perhaps by influencing selective attention to cues, even when participants were told to disregard such knowledge, and clearly successfully achieved this when judging causal relationships. These findings imply that instructing participants to ignore prior knowledge in a familiar scenario may not be entirely effective, learning is biased by prior knowledge even when given instructions to disregard it, thus researchers must consider the impact of pre-existing or commonly held beliefs on the relationships in the task.

## Data Availability Statement

The datasets presented in this study can be found in the Open Science Framework at: https://osf.io/t68rk/.

## Ethics Statement

The studies involving human participants were reviewed and approved by the Human Research Ethics Committee of the University of Sydney. The patients/participants provided their written informed consent to participate in this study.

## Author Contributions

JG and EL contributed to the conception and design of the study and the drafting and revising of the manuscript. JG collected and analyzed the data. Both the authors contributed to the article and approved the submitted version.

### Conflict of Interest

The authors declare that the research was conducted in the absence of any commercial or financial relationships that could be construed as a potential conflict of interest.

## Footnotes

^1^Mauchly’s test of sphericity indicated that the assumption of sphericity was violated. We therefore report the Greenhouse-Geisser corrected degrees of freedom for this ANOVA.

^2^The high vs. low comparison involves comparing the average of four cues (from the high conditions) against the average of two (from the low conditions). In principle, it might be possible that this difference alone introduces a bias in the results (*via* regression to the mean, for instance). To allay concerns about this, we conducted a bootstrapping exercise that randomly shuffled scores into four vs. two conditions separately for each instruction group and each test measure (learning scores and causal ratings). If there were bias as a result of comparing the mean of four cues to the mean of two, the mean of the differences from the randomly shuffled distributions of four and two cues should differ significantly from zero. The mean differences for learning scores and causal ratings for each instruction group ranged from -0.082 to 0.024. None of these differed significantly from zero, the largest *t*(9999) = 1.549, *p* = 0.121, and Bayes factors all indicated a strong support for the null hypothesis (smallest *BF_01_* = 26.73). The low – high learning score effect illustrated in Figure 2B was more extreme than 99.56% of the bootstrapped values, and the low – high causal rating effect for the use group illustrated in Figure 3B was more extreme than 100% of the bootstrapped values.
