# Supplementary material for: Can We Set Aside Previous Experience in a Familiar Causal Scenario?
Source: Front Psychol. 2020 Nov 30;11:578775. doi: 10.3389/fpsyg.2020.578775 (PMC7734345; doi:10.3389/fpsyg.2020.578775)
Supplement: Supplementary file 1 [file Data_Sheet_1.pdf]

Supplementary Material

1 Survey results

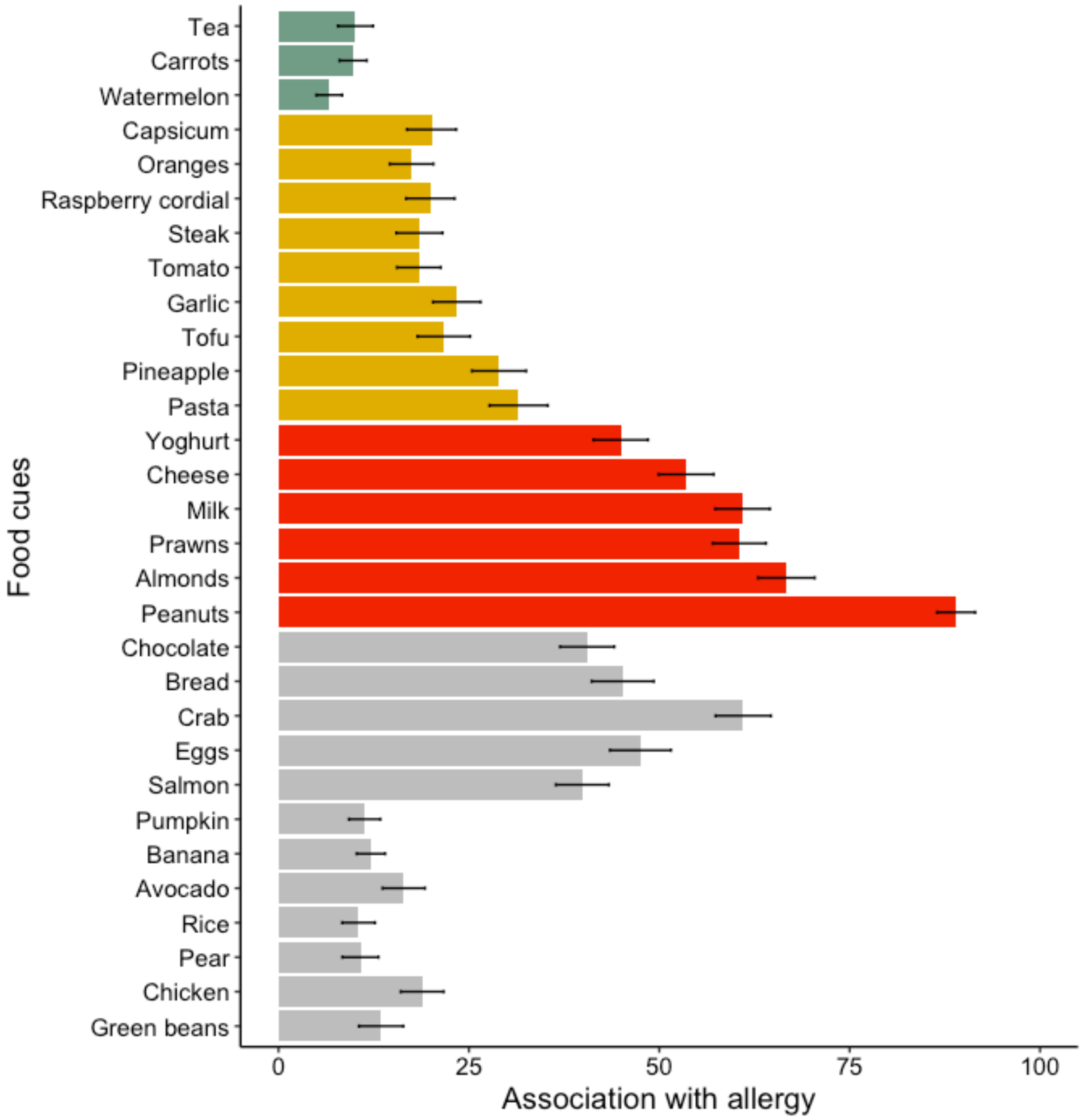

Supplementary Figure 1. Ratings of association with allergy from an initial survey of an independent sample. Bars represent the mean rating of association with allergy for each food item included in the survey. Foods in grey were not selected for the study. The colours represent the allergenic category foods were classified into for the main study: high allergenic foods in red, moderately allergenic foods in orange, and low allergenic foods in green.

Supplementary Table 1

Proportion of sample reporting specific symptoms in the initial survey.

	Allergenic symptoms by category			
Food item	N/A	Anaphylactic	Gastrointestinal	Other
Peanuts	0.01	**0.74**	0.33	0.30
Almonds	0.04	**0.61**	0.32	0.22
Prawns	0.04	**0.43**	0.54	0.26
Milk	0.03	0.17	**0.75**	0.19
Cheese	0.01	0.17	**0.67**	0.20
Yoghurt	0.07	0.10	**0.70**	0.12
Pasta	0.31	0.09	0.44	0.13
Pineapple	0.28	0.23	0.24	0.14
Steak	0.43	0.09	0.27	0.08
Tomato	0.41	0.12	0.25	0.10
Tofu	0.46	0.11	0.25	0.06
Garlic	0.31	0.10	0.29	0.11
Raspberry cordial	0.37	0.06	0.24	0.14
Oranges	0.51	0.12	0.14	0.06
Capsicum	0.44	0.13	0.20	0.12
Watermelon	**0.63**	0.03	0.13	0.05
Carrots	**0.63**	0.07	0.15	0.04
Tea	**0.54**	0.07	0.13	0.13
Eggs	0.12	0.29	0.52	0.13
Green beans	0.63	0.04	0.16	0.05
Chicken	0.43	0.08	0.34	0.07
Pear	0.66	0.05	0.12	0.06
Salmon	0.21	0.21	0.46	0.17
Rice	0.63	0.04	0.18	0.07
Bread	0.13	0.15	0.49	0.10
Avocado	0.50	0.07	0.23	0.10
Pumpkin	0.59	0.03	0.21	0.04
Crab	0.09	0.38	0.54	0.24
Banana	0.56	0.07	0.21	0.05
Chocolate	0.13	0.22	0.45	0.25

Note. For simplicity, symptoms are categorised by type. For example, Anaphylactic symptoms include ‘difficulty breathing’, gastrointestinal symptoms include ‘stomach ache,’ and any that could not be classified thus, e.g. ‘headache’ were included in category Other. Foods in italics were included in the main study an in bold are the proportions used to categorise them.

2 Filler task performance

Between the training and test phase, participants completed a short filler task in which they had to assess the logical validity of a number of syllogisms. From a possible score of 8, those in the ignore group scored *M* = 5.03 (*SD* = 2.07), and those in the use group scored *M* = 5.07 (*SD* = 2.10). A Bayesian t-test revealed evidence for the null that performance on the filler task was equivalent across the instruction groups, BF_{01} = 5.20, *error%* = 0.034, *t*(122) = -0.09, *p* = 0.931, *d* = -0.015.

3 Filler cues performance

To reduce the overall base rate of allergic reactions, a number of filler compounds paired with no allergic reaction were included in the design (see Table 1). Participants showed clear evidence of learning these contingencies, mean proportion correct predictions for these cues in the final block of training was greater than 98% in both instruction groups. The mean learning scores and causal ratings for the filler cues are reported below in Table S2.

Supplementary Table 2

Learning scores and causal ratings for filler cues

	Learning scores		Causal ratings	
	Ignore	Use	Ignore	Use
High (C/F)	82.08 (29.28)	71.19 (39.88)	30.27 (31.47)	27.76 (26.20)
Low (J)	84.78 (40.51)	85.42 (33.22)	16.22 (30.18)	19.46 (27.81)
Paired with high (T/W)	74.50 (35.22)	76.03 (39.21)	26.50 (31.80)	19.83 (23.20)
Paired with low (Z)	86.16 (40.22)	87.02 (36.40)	17.40 (31.78)	19.78 (27.65)
